# Are acute type A aortic dissections atherosclerotic?

**DOI:** 10.3389/fcvm.2022.1032755

**Published:** 2023-01-09

**Authors:** Nimrat Grewal, Onur Dolmaci, Evert Jansen, Robert Klautz, Antoine Driessen, Jan Lindeman, Robert E. Poelmann

**Affiliations:** ^1^Department of Cardiothoracic Surgery, Amsterdam University Medical Center, Amsterdam, Netherlands; ^2^Department of Cardiothoracic Surgery, Leiden University Medical Center, Leiden, Netherlands; ^3^Department of Anatomy and Embryology, Leiden University Medical Center, Leiden, Netherlands; ^4^Department of Vascular Surgery, Leiden University Medical Center, Leiden, Netherlands; ^5^Institute of Biology, Animal Sciences and Health, Leiden University, Leiden, Netherlands; ^6^Department of Cardiology, Leiden University Medical Center, Leiden, Netherlands

**Keywords:** type A aortic dissection, cardiovascular risk, atherosclerosis, embryology, intima

## Abstract

**Background:**

Type A aortic dissections (TAAD) are devastating aortic complications. Patients with Marfan syndrome, a bicuspid aortic valve or a thoracic aortic aneurysm have an increased risk to develop a TAAD. These predisposing conditions are characterized by a histologically thin intimal layer and hardly any atherosclerosis. Little is known about the susceptibility for atherosclerosis in patients with a type A aortic dissection.

**Objective:**

We aim to systematically describe atherosclerotic lesions in TAAD patients.

**Materials and methods:**

A total of 51 patients with a TAAD (mean age 62.5 ± 10.8 years, 49% females) and 17 control patients (mean age 63 ± 5.5 years, 53% females) were included in this study. Cardiovascular risk factors were assessed clinically. All sections were stained with Movat pentachrome and hematoxylin eosin. Plaque morphology was classified according to the modified AHA classification scheme proposed by Virmani et al.

**Results:**

In the TAAD group thirty-seven percent were overweight (BMI > 25). Diabetes and peripheral arterial disease were not present in any of the patients. Fifty-nine percent of the patients had a history of hypertension. The intima in TAAD patients was significantly thinner as compared to the control group (mean thickness 143 ± 126.5 μm versus 193 ± 132 μm, *p* < 0.023). Seven TAAD patients had a normal intima without any form of adaptive or pathological thickening. Twenty-three TAAD patients demonstrated adaptive intimal thickening. Fourteen had an intimal xanthoma, also known as fatty streaks. A minority of 7 TAAD patients had progressive atherosclerotic lesions, 4 of which demonstrated pathological intimal thickening, 3 patients showed early fibroatheroma, late fibroatheroma and thin cap fibroatheroma. In the control group the majority of the patients exhibited progressive atherosclerotic lesions: three pathologic intimal thickening, two early fibroatheroma, six late fibroatheroma, one healed rupture and two fibrotic calcified plaque.

**Discussion:**

This study shows that TAAD patients hardly exhibit any form of progressive atherosclerosis. The majority of TAAD patients showcase non-progressive intimal lesions, whereas the control group mostly demonstrated progressive intimal atherosclerotic lesions. Findings are independent of age, sex, or the presence of (a history of) hypertension.

## 1. Introduction

A type A aortic dissection is a life-threatening condition caused by a tear in the intimal layer, which allows blood to surge through the middle layer of the wall, causing the vascular layers to separate (dissect) from one another. Epidemiologically, patients with hypertension, connective tissue disorders or a bicuspid aortic valve (BAV) are at highest risk to develop a thoracic aortic aneurysm or aortic dissection (TAAD) (1, 2). Traditionally, cardiovascular risk factors such as aging, dyslipidaemia, smoking, chronic kidney disease, and diabetes were related to the occurrence of TAADs. Thoracic aortic aneurysms and dissections can, however, develop in the absence of cardiovascular risk factors and affect young individuals as a result of genetic disorders (3, 4). The mechanisms initiating and stimulating the progression of an aortic dissection are still poorly understood. More recently attention has been drawn toward the intrinsic pathology of the aortic vessel wall. Bicuspid aortic valve and Marfan syndrome (MFS) are both associated with an extremely high risk to develop a thoracic aortic dissection (5, 6). During the last decade, a number of studies dealing with the embryonic development of aortopathy in BAV and MFS have significantly improved our knowledge on the etiology (7, 8). In both conditions an altered neural crest cell and second heart field contribution, separately or in combination, can account for a maturation defect of the vascular smooth muscle cells resulting in a structurally different aortic root and ascending aortic wall (8, 9). Besides undifferentiated vascular smooth muscle cells in the medial layer, significant differences in the intimal layer have also been noted in BAV and MFS. The intima has been found significantly thinner in the BAV and MFS patient groups as compared to individuals with a tricuspid aortic valve (TAV). Vascular smooth muscle cells and smooth muscle cell-derived cells are a major source of plaque cells and extracellular matrix at all stages of atherosclerosis (10). Clinically, both BAV and MFS are characterized by significantly less atherosclerosis. Findings which are in line with earlier studies highlighting that thoracic aortic aneurysms are associated with decreased systemic atherosclerosis (11). We postulate that the combination of a thin intimal layer and a defect in vascular smooth muscle cell differentiation might act protective for the development of atherosclerosis in the vessel wall. Less is, however, known about the role of atherosclerosis in patients with a type A aortic dissection. Recently we concluded that patients with a type A aortic dissection also demonstrate a significantly thinner intimal layer, even in the absence of a genetic syndrome or a bicuspid aortic valve (12). Considering the overlap in pathogenetic mechanisms between BAV, MFS, and type A aortic dissections it is particularly interesting to study the presence of atherosclerosis in aortic dissections.

Earlier studies have suggested that patients with a type A aortic dissection are less prone for atherosclerosis, however, this has been concluded on basis of imaging studies (11, 13). The novelty of this paper is that we study atherosclerosis on tissue level and can detect early stages of atherosclerosis which would be missed by only studying the images of transesophageal echocardiography or computed tomography images (11, 14). The aim of this study is to systematically classify atherosclerotic lesions in the type A aortic dissection population according to the adapted AHA classification as proposed by Virmani et al. (15).

## 2. Materials and methods

### 2.1. Patients and tissue samples

Fifty-one ascending aortic wall samples were obtained from the aortotomy site as residual aortic wall material during an ascending aortic replacement in patients with an acute type A aortic dissection at the Leiden University Medical Center, Leiden, Netherlands. Patients with a proven genetic disorder (e.g., Marfan disease) or a bicuspid aortic valve were excluded. Seventeen control aortas were obtained during post-mortem autopsies.

Sample collection was uniform in all patients: ascending aortic wall specimen were obtained from the aortotomy site. The aortotomy site is classically in the middle of the ascending aorta, just beneath the pericardial fold. Circular tissue was obtained and embedded in paraffin. The complete circular ascending aortic wall was sectioned to avoid sampling of the aortic tissue.

Sample collection and handling was carried out according to the official guidelines of the Medical Ethical Committee of Leiden University Medical Center and the code of conduct of the Dutch Federation of Biomedical Scientific Societies.^1^ Autopsy has been performed according to the guidelines of the pathology department. Tissue collection was performed according to the regulations and protocols for secondary tissue use of the department of pathology at the Leiden University Medical Center. Written informed consent was obtained.

After excision, specimens were fixed in 4% formalin (24 h), decalcified in a formic acid-formate buffer (120 h), and embedded in paraffin. Transverse sections (4 μm) were mounted on precoated Starfrost slides (Klinipath BV, Duiven, Netherlands).

### 2.2. Histological classification of the lesions

The sectioning and staining protocols have been described previously (16). Hematoxylin-eosin (HE) and Movat pentachrome staining was performed for the histological evaluation of all samples of each circular aortic specimen. Sections were studied with a Leica BM5000 microscope equipped with plan achromatic objectives (Leica Microsystems, Wetzlar, Germany). Each section was individually classified according to the modified classification of the AHA as proposed by Virmani et al. (15), by two independent observers with no knowledge of the characteristics of the aortic patch (Table 1). Across the circular ascending aortic wall tissue the plaque with the most advanced grade of atherosclerosis according to Virmani et al. (15) called the “dominant plaque” was analyzed for this study.

**TABLE 1:** Atherosclerotic lesions in type A aortic dissection patients.

Morphological description	Associated AHA classification	*N*	Mean age	Female (%)
Normal aorta	–	7	63.3 ± 9.3	42.9%
**Non-progressive intimal lesions**				
Adaptive intimal thickening	I	24	62.1 ± 10	61%
Intimal xanthoma	II	13	62.6 ±	14%
**Progressive atherosclerotic lesions**				
Pathological intimal thickening	III	4	64.2 ± 7	100%
Early fibroatheroma	IV	1	51	100%
Late fibroatheroma	IV/V_a_	1	65	100%
Thin-cap fibroatheroma	–	1	66	0%
Plaque rupture	VI	0	–	–
Healing rupture	VI	0	–	–
Fibrotic calcified plaque	V_b,c_, VII	0	–	–

Given a role of inflammation in atherosclerosis we also studied the presence of inflammatory infiltrates in the intimal layer of type A aortic dissection and control patients. As inflammatory cells are acute phase proteins, an abundance of these cells is encountered in the dissected medial layer. To study inflammatory cells associated with intimal atherosclerosis, we studied the influx of inflammatory cells in the intimal layer using the HE and MOVAT pentachrome stained sections, indexed from zero (no inflammatory cells), 1 (a few cells), 2 (groups of cells) to 3 (large clusters of cells).

### 2.3. Statistical analysis

All numerical data are presented as the mean ± standard deviation. Statistical differences were evaluated with the Mann–Whitney U test for comparison between the groups. One, two and three way ANCOVA, binary logistic regression and linear regression analysis were performed to correct for age, sex and hypertension. Significance was assumed when *p* < 0.05 with SPSS 26.0 (SPSS Inc., Chicago, IL, USA) was used for the statistical analyses.

## 3. Results

### 3.1. Patient population

A total of 51 patients with a type A aortic dissection were included in the study. The mean age of the study population was 62.5 ± 10.8 years. Females and males were evenly distributed in the study group (49% females). All patients had a tricuspid aortic valve and no underlying aortic genetic disease was identified after genetic screening by the institutional geneticist.

The mean body-mass index of all patients was 26.01 kg/m^2^; 19 patients (37%) were considered overweight (BMI > 25). Smoking status was not known for most patients. Diabetes and peripheral arterial disease were not present in any of the patients. Chronic obstructive pulmonary disease was seen in one patient. Fifty-nine percent of the patients had a history of hypertension (antihypertensive medication or systolic blood pressure > 140 mmHg and diastolic > 90 mmHg in the period).

The control group of 17 patients were obtained post-mortem. The mean age of the control patients was 63 ± 5.5 years. Females and males were evenly distributed in the controls (53% females). All control patients had a non-cardiac cause of death, had a tricuspid aortic valve and no underlying genetic disease was known. Data on cardiovascular risk factors could not be retrieved for all control patients.

### 3.2. Morphometric measurements

**FIGURE 1:**
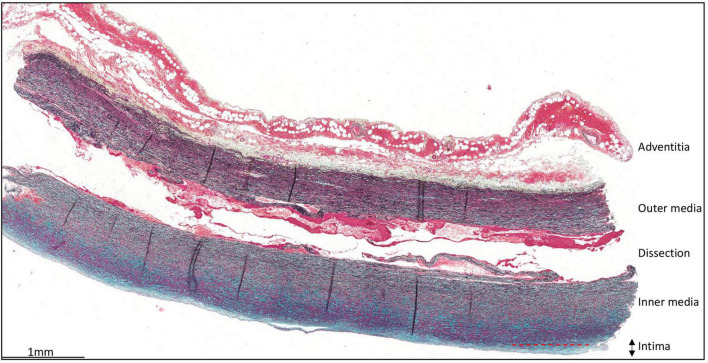
Transverse histologic section (4 μm) of a type A aortic dissection aortic specimen. The vessel wall layers are indicated: adventitia, media, and intima. The intimal layer is defined as the area between the endothelial cells lining the luminal surface and the lamina elastica interna. Scale bar shown in figure.

The intima is defined as the area between the inner surface of the aortic wall, lined by the endothelial cells and the first elastic lamellae of the media (Figure 1). The mean intimal thickness in all type A dissection patients was 143 ± 126.5 μm. No significant difference in intimal thickness was observed when corrected for age and sex or in the presence of (a history of) hypertension (Age vs intimal thickness *p* = 0.406; sex vs intimal thickness: OR 1.00 (95% CI 1.00–1.01), *p* = 0.669; Hypertension vs intimal thickness OR 1.00 (95% CI 1.00–1.00), *p* = 0.860). In control patients the mean intimal thickness was 193 ± 132 μm, which is significantly thicker as compared to the type A aortic dissection patients (*p* < 0.023).

### 3.3. Non-progressive intimal lesions in type A aortic dissection patients

**FIGURE 2:**
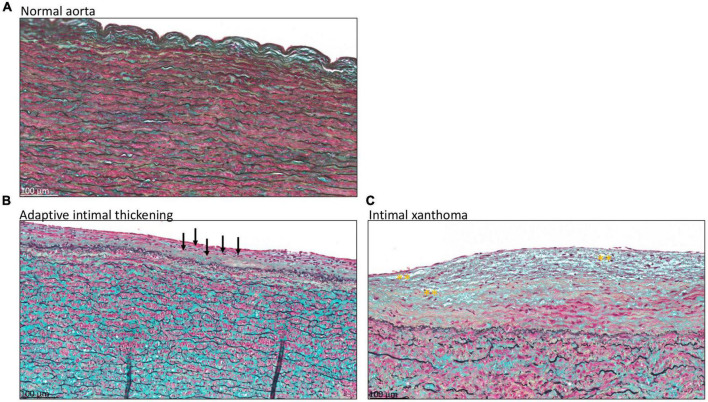
Transverse histologic section of type A aortic dissection aortic specimen (4 μm), stained with MOVAT pentachrome. **(A)** Shows a normal intimal layer, without any signs of adaptive or pathological thinning. In panel **(B)** an adaptive thickened intima is seen with mainly vascular smooth muscle cells in the proteoglycan-rich matrix, a few of which are depicted with black arrows. **(C)** Intimal xanthoma is seen in a patient with macrophage derived foam cells in the matrix, highlighted with yellow asterisks. Scale bar shown in the figures.

Atherosclerotic lesions in the intima were scored according to the modified classification of the AHA as proposed by Virmani et al. (15), Table 1. Seven patients had a normal intima without any form of adaptive or pathological thickening (Figure 2A). The intimal layer had a mean thickness of 81 ± 62.7 μm in these patients. Mean age in this group was 63.3 ± 9.3 years and 43% was female.

Twenty-three type A aortic dissection patients demonstrated adaptive intimal thickening (Figure 2B). In these patients the intimal thickening consisted mainly of smooth muscle cells with an increase in proteoglycan-rich matrix. Mean intimal thickness in this group was 103.4 ± 56 μm, and mean age was 62.1 ± 10 years. Sixty-one percent of the patients with adaptive intimal thickening were female.

As soon as macrophage-derived foam cells become evident in the intima the lesions are referred to as intimal xanthoma, also known as fatty streaks (Figure 2C). These lesions were seen in 14 patients, with a mean age of 62.6 ± 14.9 years and 14% was female. Mean thickness of the intima was 151.6 ± 94.4 μm.

### 3.4. Progressive atherosclerotic lesions in type A aortic dissection patients

**FIGURE 3:**
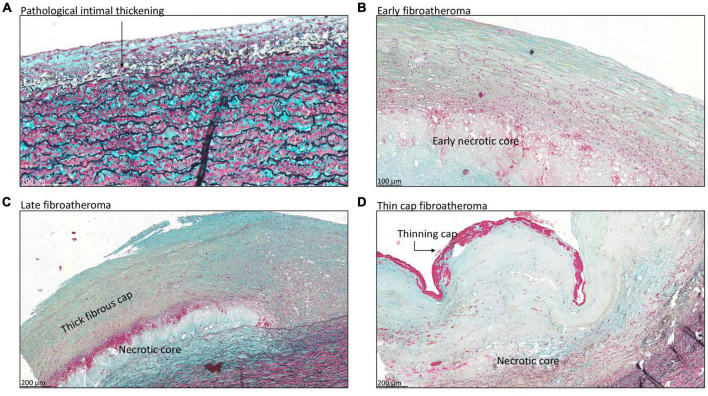
Transverse histologic section (4 μm) of type A aortic dissection aortic specimen, stained with MOVAT pentachrome. **(A)** Shows pathological intimal thickening, characterized by the presence of lipid pools deep within the intima near the intimal medial border with overlying vascular smooth muscle cells (black arrow). Fibroatheroma is seen in panels **(B–D)**, with an early necrotic core in panel **(B)**, thick fibrous cap in panel **(C)**, and thinning of the fibrous cap in panel **(D)**. Scale bar shown in the figures.

A total of seven patients had progressive atherosclerotic lesions (Figure 3 and Table 1). The mean age of these patients was 62.7 ± 7.2 years and 86% was female. Four out of these seven patients demonstrated pathological intimal thickening with a mean intimal thickness of 244.0 ± 156.2 μm (Figure 3A). Mean age of the four patients was 64.2 ± 7 years, all of which were female. The remaining three patients showed early fibroatheroma (Figure 3B), late fibroatheroma (Figure 3C) and thin cap fibroatheroma (Figure 3D).

The severity of the atherosclerotic lesions was independent of age, sex, and the cardiovascular risk factor hypertension (age vs atherosclerosis classification *p* = 0.976; sex vs atherosclerosis classification: OR 0.99 (95% CI 0.63–1.56), *p* = 0.960; hypertension vs atherosclerosis classification: OR 1.14 (95% CI 0.72–1.78); *p* = 0.587).

### 3.5. Atherosclerotic lesions in control patients and intimal inflammation

**FIGURE 4:**
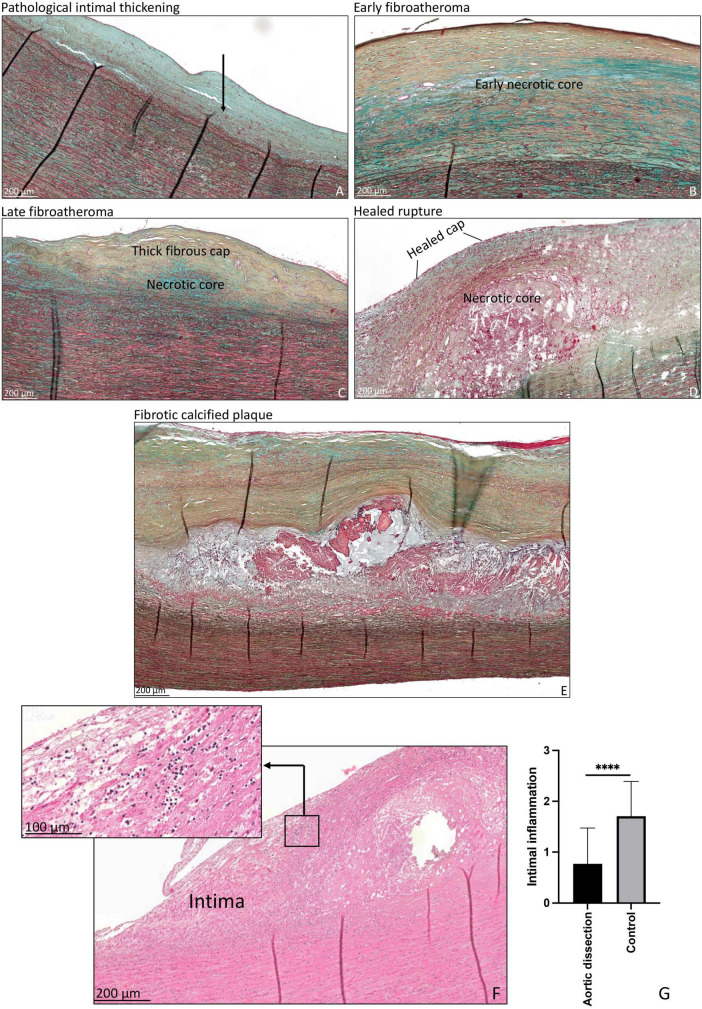
Transverse histologic section (4 μm) of control aortic specimen, stained with MOVAT pentachrome **(A–E)** and hematoxylin eosin **(F)** staining. **(A)** Shows pathological intimal thickening, lipid pools are seen deep within the intima near the intimal medial border with overlying vascular smooth muscle cells (black arrow). **(B)** Shows early fibroatheroma, with an early necrotic core. **(C)** Shows late fibroatheroma with a thick fibrous cap overlying the necrotic core. Healed rupture is seen in panel **(D)** and a fibrotic calcified plaque in panel **(E)**. Intimal inflammation is seen in panel **(F)**, the insert demonstrates a detail of the inflammatory cells. The qualitative amount of inflammatory cells was significantly higher in the control group as compared to the type A aortic dissection patients (*p* < 0.0001) **(G)**. ****p < 0.0001. Scale bar shown in the figures.

Non-progressive atherosclerotic lesions (intimal xanthoma) were seen in only three patients (Table 2). Patients from the control group predominantly exhibited progressive atherosclerotic lesions. Three patients showed pathological intimal thickening (Figure 4A), two had early fibroatheroma (Figure 4B), six demonstrated late fibroatheroma (Figure 4C), one had a healed rupture (Figure 4D), and two had a fibrotic calcified plaque (Figure 4E and Table 2). Sex was randomly distributed across the non- and progressive atherosclerotic lesions.

**TABLE 2:** Atherosclerotic lesions in control patients.

		Type A dissections group	Control group
**Morphological description**	**Associated AHA classification**	***N***	***N***
Normal aorta	–	7 (14%)	0
**Non-progressive intimal lesions**			
Adaptive intimal thickening	I	24 (47%)	0
Intimal xanthoma	II	13 (25%)	3 (18%)
**Progressive atherosclerotic lesions**			
Pathological intimal thickening	III	4 (8%)	3 (18%)
Early fibroatheroma	IV	1 (2%)	2 (12%)
Late fibroatheroma	IV/V_a_	1 (2%)	6 (35%)
Thin-cap fibroatheroma	–	1 (2%)	0
Plaque rupture	VI	0	0
Healing rupture	VI	0	1 (6%)
Fibrotic calcified plaque	V_b,c_, VII	0	2 (12%)

As inflammatory cells are associated with the development of atherosclerosis, we studied the infiltration of inflammatory cells in the intimal layer. The qualitative amount of inflammatory cells was significantly higher in the control group as compared to the type A aortic dissection patients (*p* < 0.0001) (Figures 4F, G).

## 4. Discussion

In this study we analyzed atherosclerotic lesions in type A aortic dissection patients. We found a very low incidence of progressive atherosclerotic lesions in the study population. Major cardiovascular risk factors for atherosclerosis (i.e., age, sex, hypertension, and diabetes mellitus) were not associated with atherosclerosis. The control group on the other hand demonstrated predominantly progressive atherosclerotic lesions. The intimal layer further showed significantly more influx of inflammatory cells in the control patients as compared to the intimal layer in type A aortic dissections, this finding is in line with earlier suggestions that inflammation may contribute to the development of atherosclerosis. The presence of inflammatory cells in the intimal layer of the ascending aorta is an interesting finding and further studies should be performed to characterize the type of inflammatory cells present. Findings of this study are consistent with previous observations that conditions predisposing for the development of a type A aortic dissection, being patients with Marfan syndrome, a bicuspid aortic valve and a thoracic aortic aneurysm, also associate with a lower prevalence of atherosclerosis (11, 17).

A type A aortic dissection is a life-threatening aortic complication. Although several risk factors have been described predisposing individuals for this lethal condition, the exact pathogenesis has not yet been identified (18). Roughly, the development of an aortic dissection requires three main pathological conditions, being mechanical wall stress, a susceptible intimal layer and medial degeneration (12, 19). Our previous study had shown that medial pathology in type A aortic dissections is comparable with thoracic aneurysms in patients with a tricuspid aortic valve with smooth muscle cell nuclei loss, mucoid extracellular matrix accumulation and elastic fiber fragmentation and loss (12). The intimal layer was, however, significantly thinner as compared to the tricuspid thoracic aortopathy patients, resembling the intima in patients with Marfan syndrome and a bicuspid aortic valve (20, 21). As aortic dissections commence with a tear in the intimal layer, it is particularly interesting to understand whether the pathology present is comparable to degenerative or genetic thoracic aortopathy.

Age is an important factor in the atherosclerotic process and is related with a significant change in the physiological and pathological properties of the vessel wall (22, 23). Studies focusing on the natural history of atherosclerosis have shown that advanced atherosclerotic disease is common in people over 45. In this study we excluded patients with a genetic syndrome and/or a bicuspid aortic valve, and all patients analyzed were aged and presented with so-called degenerative thoracic aortopathy. In the study population age did not differ significantly between the sub-groups of non-and progressive intimal lesions as suggested by Virmani et al. (15). Moreover, 86% of the patients had no signs of progressive intimal atherosclerosis, with the majority demonstrating a normal aorta or adaptive intimal thickening even at an advanced age. This is contrast to the control group of comparable age and gender distribution, which predominantly exhibited progressive atherosclerotic lesions. A comparative study performed by Heng et al. used heart transplant recipients as control aortic tissue and found no atherosclerosis in this group (24). These patients were, however, almost 5–6 years younger than the patients studied in our paper. This could explain the difference in our findings. Future studies should pay additional attention to the cardiovascular risk factors in the control group. Our data further suggests that in the type A dissection population progressive aortic atherosclerosis occurs more often in women than in men, an observation that concurs with clinical observations showing that peripheral artery disease develops earlier and at a relatively high rate in women (25, 26). In the control group a female predominance was not observed in the progressive aortic atherosclerotic lesions.

Earlier studies have suggested that patients with a thoracic aortic aneurysm or aortic dissection demonstrate less atherosclerotic lesions (11, 13, 17, 27). These studies used imaging modalities such as TEE or CT scanning to score atherosclerotic lesions across the aorta (28). In this paper we studied the histological classification of atherosclerosis on aortic tissue level to be able to also detect early stages of atherosclerosis which would be missed by only studying the images of transesophageal echocardiography or computed tomography images (11, 14). As more than 90% of the type A aortic dissections commence within the first few centimeters of the ascending aortic wall, and the aortic root and arch are not always involved, we uniformly studied the ascending aorta in all patients. In our earlier studies we have paid particular attention to the role of shear stress on the histopathology of the aortic wall in patients with a bicuspid and tricuspid aortic valve, with and without dilatation and found no difference in the amount of atherosclerosis (29). With magnetic resonance imaging the area with maximum shear stress in the ascending aortic wall was compared to the opposite site (non-jet side). Differences were appreciated in the intimal thickness, but without features of atherosclerosis (29).

We recognize that atherosclerosis is a pathological entity, even though our study has dealt with many limitations by performing histopathological analysis and avoiding sampling errors, the lesions represent a very short segment of ascending aorta, and areas of atheroma may be missed. Staining for smooth muscle cells and immune cell markers should be considered in future studies to confirm our findings. Our study is further limited by the low number of control patients versus the dissected specimen. Even though genetic screening is performed in the patients to exclude an aortic genetic disease, unknown genetic mutations associated with sporadic type A dissections can be missed.

Our findings emphasize that a type A aortic dissection occurs in the absence of clinical cardiovascular risk factors as diabetes, dyslipidaemia and peripheral arterial and despite aging most patients have minimal atherosclerotic lesions. We postulate that a thin intimal layer could increase susceptibility for a future aortic dissection, but at the same time acts protective for systemic atherosclerosis.

## Data availability statement

The raw data supporting the conclusions of this article will be made available by the authors, without undue reservation.

## Ethics statement

The studies involving human participants were reviewed and approved by the Medical Ethical Committee of Leiden University Medical Center. The patients/participants provided their written informed consent to participate in this study.

## Author contributions

NG conceived and designed the experiments, performed the experiments, analyzed and interpreted the data, contributed reagents and materials, and wrote the manuscript. OD, EJ, and AD wrote the manuscript. RK conceived and designed the experiments, contributed reagents and materials, and wrote the manuscript. JL contributed reagents and materials and wrote the manuscript. RP conceived and designed the experiments, analyzed and interpreted the data, contributed reagents and materials, and wrote the manuscript. All authors contributed to the article and approved the submitted version.

## Footnotes

1
www.FMWV.nl
